# Hemodynamic Analysis in Aortic Dilatation after Arterial Switch Operation for Patients with Transposition of Great Arteries Using Computational Fluid Dynamics

**DOI:** 10.1007/s12265-024-10562-2

**Published:** 2024-09-25

**Authors:** Woo Young Park, Sang Yun Lee, Jongmin Seo

**Affiliations:** 1 Department of Pediatrics, Seoul National University Children’s Hospital, Seoul National University College of Medicine, 101, Daehak-ro, Jongno-gu, Seoul, Republic of Korea; 2 Department of Mechanical Engineering, Kyung Hee University, 1732 Deogyeong-daero, Giheung-gu, Yongin-si, Gyeonggi-do Republic of Korea

**Keywords:** Transposition of the great arteries, Arterial switch operation, Aortic root dilatation, Computational fluid dynamics, Wall shear stress, Vorticity

## Abstract

After an arterial switch operation for complete transposition of the great arteries, neo-aortic root dilatation occurs, with unclear hemodynamic effects. This study analyzes three groups (severe dilation, mild dilation, and normal) using computational fluid dynamics (CFD) on cardiac CT scans. Aortic arch angles in severe (median 72.3, range: 68.5–77.2) and mild dilation (76.6, 71.1–85.2) groups are significantly smaller than the normal group (97.3, 87.4–99.0). In the normal and mild dilatation groups, Wall Shear Stress (WSS) exhibits a consistent pattern: it is lowest at the aortic root, gradually increases until just before the bend in the aortic arch, peaks, and then subsequently decreases. However, severe dilation shows disrupted WSS patterns, notably lower in the distal ascending aorta, attributed to local recirculation. This unique WSS pattern observed in severely dilated patients, especially in the transverse aorta. CFD plays an essential role in comprehensively studying the pathophysiology underlying aortic dilation in this population.

**Graphical Abstract:**

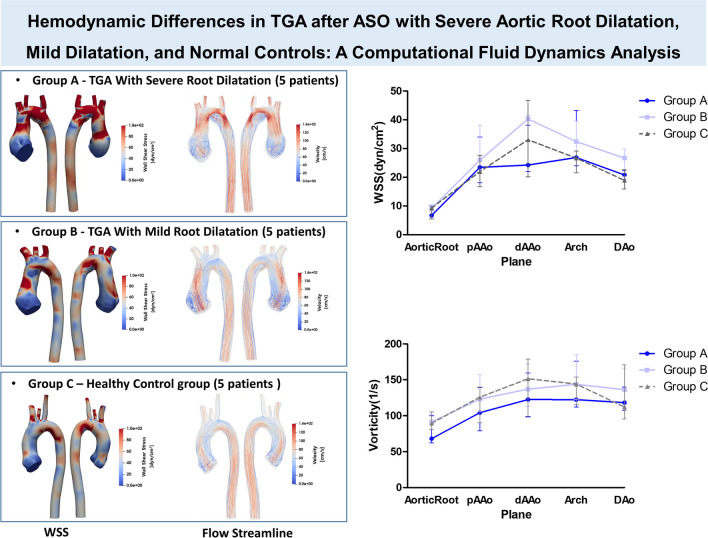

**Supplementary Information:**

The online version contains supplementary material available at 10.1007/s12265-024-10562-2.

## Introduction

Complete transposition of the great arteries (TGA) is one of the most common cyanotic heart defects which accounts for three percent of all congenital heart disease and twenty percent of cyanotic heart disease [1]. The arterial switch operation (ASO) is the current operation of choice for patients with TGA [2]. The ASO has shown excellent early and long-term survival and good left ventricle systolic function outcomes into adult life in most of patients. However, studies on large cohorts, such as one study involving 844 patients, have shown progressive neo-aortic root dilation over time, with an associated increased incidence of neo-aortic regurgitation and the need for root and/or valve reoperation [3, 4]. Therefore, numerous studies have been conducted to elucidate the factors implicated in the pathophysiological processes underlying the dilation of the neo-aortic root, such as anatomical variations, surgical techniques, and hemodynamic changes post-ASO. Despite these efforts, our understanding remains largely incomplete [5–12].

Computational fluid dynamics (CFD) employs numerical methods and high-performance computing to predict fluid flow phenomena, relying on the principles of conservation laws governing fluid dynamics, including mass, momentum, and energy conservation equations [13]. CFD provides quantitative visualizations of blood flow, pressure gradients, vessel wall deformations, and shear stress distributions on the vessel wall [14]. The significance of CFD in this study lies in its ability to provide detailed hemodynamic insights that are not possible through conventional imaging or clinical assessments alone. By simulating blood flow dynamics, CFD can help identify specific patterns of wall shear stress (WSS) and vorticity that may contribute to or result from neo-aortic root dilation. These insights are crucial for understanding the progression of dilation and for developing targeted interventions [15].

Previous studies using CFD in patients with TGA after ASO have only compared the surgical group to the normal control group [4, 16]. However, no study has examined the differences between TGA patients who underwent ASO and experienced severe neo-aortic root dilation and those with mild neo-aortic root dilation in comparison to normal subjects. Analyzing the variations between these patient groups is essential for understanding their impact on aortic dilation. Therefore, the aim of this study to assess the aortic blood flow patterns, aortic WSS, and blood vorticity in three distinct groups: patients with TGA after ASO experiencing severe neo-aortic root dilation (Group A), those with mild neo-aortic root dilation (Group B), and a normal control group (Group C). Our study is, to the best of our knowledge, the first to apply CFD to investigate altered hemodynamics in TGA patients who underwent ASO, stratified into severe dilatation, mild dilatation, and normal groups for advanced treatment planning after ASO.

## Methods

### Study Population

This study is a retrospective study conducted on patients diagnosed with complete TGA who underwent ASO at Seoul National University Children’s Hospital from January 1, 2003, to December 31, 2013. Our analysis involves cardiac computed tomography (CT) images of patients who received cardiac CT scans between the ages of 8 and 18 within this patient cohort. The selected age range of 8 to 18 years ensures the inclusion of long-term follow-up data, as neo-aortic root dilatation is primarily a long term complication post-ASO [3, 4]. Additionally, we included patients aged 8 years and older because they are more likely to have a body weight of at least 30 kg and a body surface area (BSA) of at least 1.2 m^2^, approximating adult body size. The upper limit of 18 years is based on the reference population used by Lopez et al. for Z-score calculations [17]. The patient groups are categorized into three groups: severe aortic dilation (Z-score ≥ 4) (Group A), mild aortic dilation (2 < Z-score < 4)(Group B), and a healthy control group (Z-score $\le 2)$(Group C) consisting of adolescents aged between 8 and 18 who underwent cardiac CT scans due to chest pain but exhibited no structural or functional abnormalities. Following the age-matching process, the complete cohort consisted of fifteen patients, divided into three groups: 5 individuals in Group A, 5 in Group B, and 5 in Group C. This study excluded patients who underwent ASO after the diagnosis of TGA and subsequently deceased during the study period, as well as those with combined arch anomalies requiring staged operations. This study was approved by the Institutional Review Board of the Seoul National University Hospital (IRB number: H-2311–002-1481).

### Size Measurement

**Fig. 1:**
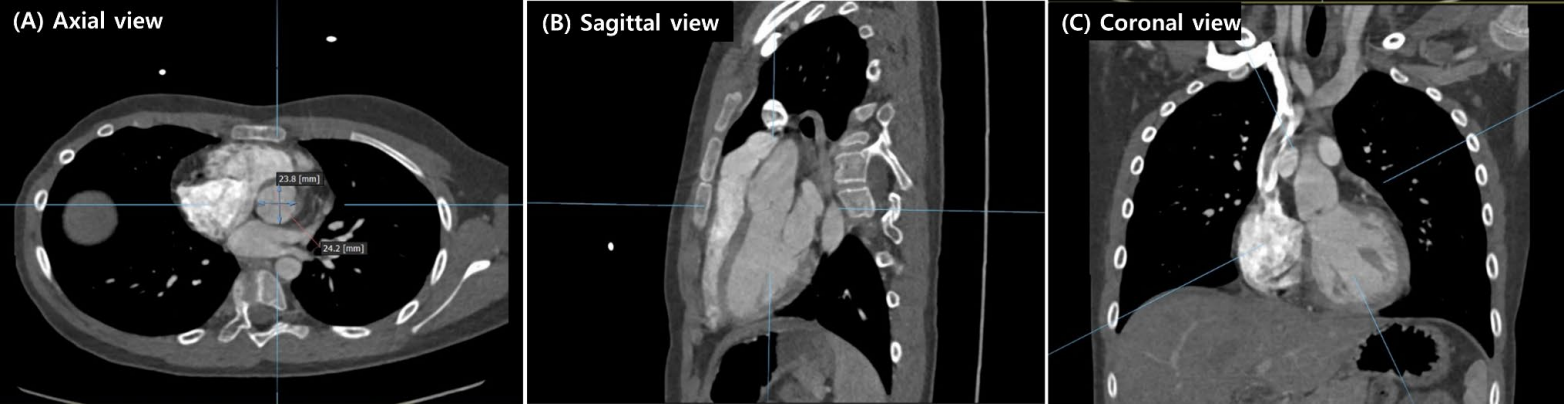
Measurement of the diameter of the neo-aortic valve at the annulus level. For the precise selection of the accurate hinge point and level, axial (**A**), sagittal (**B**), and coronal (**C**) views in two-dimensional images were simultaneously utilized

**Fig. 2:**
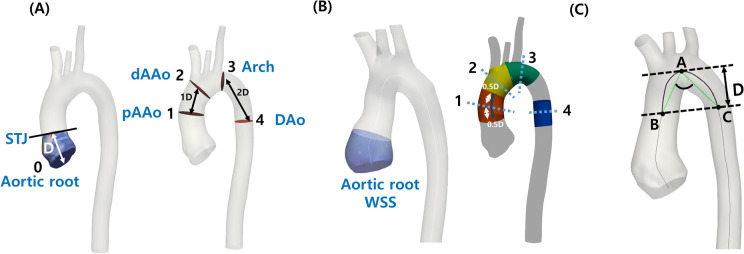
Measurement of wall shear stress, vorticity, and aortic arch angle. **A** Two-Dimensional Slices at Different Aortic Locations. Two-dimensional slices were obtained perpendicular to the centerline of the aorta model at five locations. Plane 0 (Aortic Root): Positioned one diameter away from the two-dimensional slice above the annulus. Plane 1 (Proximal Ascending Aorta): Located by moving one diameter in the inlet direction from the second cross-section. Plane 2 (Distal Ascending Aorta): Positioned just before the first bifurcation. Plane 3 (Aortic Arch): Placed just after the last bifurcation. Plane 4 (Descending Aorta): Defined by moving two diameters in the descending aorta direction from the third cross-section. Vorticities were calculated over these two-dimensional slices at each of the five different locations along the centerline. **B** Wall Shear Stress Calculation. Wall shear stresses were time-averaged over one cardiac cycle and area-averaged over the region of interest, as indicated in Fig. 2B. The region extended proximally from the STJ to a distance of one diameter for calculating the WSS in the aortic root (sky blue color). Using the planes labeled as Plane 1–4 in Fig. 2A as references, a region above and below each plane within a distance of 0.5 diameter was defined. In this space, WSS measurements were taken for the proximal ascending aorta (red), distal ascending aorta (yellow), aortic arch (green), and descending aorta (blue). **C** Measurement of the aortic arch angle involved identifying the highest point of the centerline of the aorta (Point A) and drawing a tangent line from this point. The second line was created by moving one diameter parallel to the tangent line and determining two points where the centerline intersected with this line (Point B and C). The angle was subsequently calculated between line AB and line AC. WSS: Wall shear stress

Retrospective measurements were conducted on images obtained from cardiac CT. Neo-aortic diameters were assessed at four specific levels: 1) neo-aortic valve annulus, from hinge point to hinge point; 2) neo-aortic sinus of Valsalva, from the internal edge to the internal edge; 3) neo-aortic sino-tubular junction (STJ) by internal edge to internal edge; and 4) mid-ascending aorta, at the level of the mid main pulmonary artery, from hinge point to hinge point. For precise selection of the accurate hinge point and level, we simultaneously utilized axial, coronal, and sagittal views in two-dimensional images (as shown in Fig. 1) as well as three-dimensional reconstructed images (as shown in Fig. 2). By combining these 2D image cuts and 3D reconstruction images, we ensured accurate measurement of the diameters. For the measurement of the diameter at the mid-ascending aorta level, we recorded both the minimum and maximum diameters and then calculated the average diameter. To account for the range in body size for neo-aortic measurements during 8–18 years, Z-scores for each patient were calculated using the pediatric reference recommended by Lopez et al. [17], which covers the age range from 0 to 18 years. The body surface area (BSA) was calculated using the Haycock method [18]. Severe dilation was defined as a Z-score of ≥ 4 in any component of the aortic root, encompassing the aortic valve annulus, sinus of Valsalva, sino-tubular junction, or ascending aorta. Mild dilatation was characterized by a Z-score between 2 and 4 in any of the aforementioned components of the aortic root.

### Comparison Factors

The factors influencing aortic root dilatation in patients with TGA who underwent ASO were chosen based on literature reviews and include male sex, the presence of VSD, Taussig-Bing anomaly, arch anomalies such as interrupted aortic arch or coarctation of the aorta, bicuspid neo-aortic valve, coronary artery abnormality, pulmonary artery banding before ASO, great artery size discrepancy, coronary transfer technique, neo-aortic regurgitation within 1 year after ASO, and arch angle [5–12]. In the study, due to missing preoperative size data, "great artery size discrepancy" was verified from surgical records or echocardiograms, where the pulmonary artery exceeded 1.5 times the aorta size.

### Patient-Specific Anatomic Modeling

We used cardiac CT images of TGA patients who underwent ASO, including those with severe neo-aortic root dilatation, mild neo-aortic root dilatation, as well as images from a healthy control group. The SimVascular software, an open-source tool [19], was utilized for the segmentation of aorta, construction of anatomical models, and the simulation of blood flow. The modeling and simulation steps encompassed geometry acquisition, description of boundary conditions, selection of computation type and parameters, simulation, and post-processing (e.g. visualizations) of numerical data. Initially, three-dimensional paths were generated by manually tracking the lumens of the aorta visible in the CTA images. Subsequently, the vessel underwent segmentation, determining its contour at central points. At each cross-section along the centerline, we determined which parts of the medical image depicted the object of interest, which was achieved through the level set method and threshold setting. These two-dimensional lumen segmentations were then lofted into a three-dimensional model, representing the anatomical model of aorta.

### Computational Fluid Dynamics

For the simulation of blood flow, we employed svSolver, integrated with SimVascular. This solver utilizes a stabilized finite element method with linear tetrahedral elements. The governing equations used to simulate blood flow were the incompressible Navier–Stokes equations and the continuity equation. Finite element meshes were generated using the TetGen package, an open-source tool [20], facilitated by SimVascular. The vessel wall was assumed to be rigid. The simulations produced time-resolved fields of blood velocity and pressure throughout the aorta model. In all simulations, blood was assumed to be a Newtonian fluid, with a viscosity of 0.04 dynes/cm^2^ and a density of 1.06 g/cm^3^. The non-Newtonian effects of blood, typically observed in vessels with diameters smaller than 300 μm, were not taken into consideration in this study [21]. We acknowledge that the non-Newtonian fluid model (e.g. Carreau-Yasuda model) can improve the simulation realism, however, given the smallest lumen diameters analyzed were on the order of 1 mm, we assume that the Newtonian fluid model can accurately predict the flow behaviors in the aorta where the shear rate is expected to remain above ~ 100/s. In this shear rate regime, the dynamic viscosity is largely unchanged [22, 23].

At the inlet of the aorta model (plane above the annulus), we prescribed a time-resolved aortic waveform obtained from healthy patients scaled to match the estimated cardiac output of each patient using their age and BSA [24]. Estimated cardiac output ranges 3.8 to 6 L/min depending on the patient. The Reynolds number associated in our simulation based on the cardiac output and the vessel diameter, $Re=\frac{Ud}{\nu }$ ranges between 1008– 1671, with mean ± standard deviation as 1333 ± 210. In this regime, the flow can be assumed as laminar or transitional flow.

Boundary conditions for blood flow at the outlets of the aorta, brachiocephalic arteries, and carotid arteries were imposed using an open-loop lumped parameter network model. The lumped parameter model was used to simulate the physiological hemodynamic response of downstream vessels in a volume-averaged manner and was prescribed as a boundary condition. This network employs an electrical circuit representation to model blood flow commonly referred to as 3-element Windkessel model consisting of two resistances and capacitance of distal vasculature [25]. These parameters underwent tuning to ensure that the simulations accurately reflected patient-specific clinically measured aortic pressure. Specifically, we iterate simulations with initial guess of the resistances and capacitances until it converges to the patient’s maximum and minimum pressure. The difference between the simulated and measured blood pressures in patients was 5.1% ± 3.4%. The distribution of flow among carotid arteries was determined based on the modified Murray’s law with an exponent of 2.0 [26].

For all 15 patients, a time step size of 0.001 s was used. We checked the time step convergence by decreasing the time step to 0.0005 s, which is half of the implemented time step, and verified that all the presented results were unchanged within 3% errors. At least eight cardiac cycles were simulated and the only last 1 heart cycle data were used to eliminate the initial transient effects. The number of total time steps varies between 8000 and 15000 depending on the patient. The number of time steps was determined by performing simulations until the blood pressure and other hemodynamic values converged within 1% of variabilities compared to the previous cycle for each patient.

We used high-resolution meshes to resolve detailed blood flow patterns. We applied an edge size of 0.8 mm for all patient models after conducting a mesh independence study, by subsequently increasing the number of mesh up to 8 million elements and confirming an appropriate edge size for mesh independence compared to the highest-resolution mesh. In addition, boundary layer meshing technique, which places smaller size meshes near the vessel wall, was added to accurately capture the wall shear stress. With these strategies, we achieved mesh convergence for both wall shear stress and vorticities within 10% errors compared to a refined mesh with double-sized elements. The number of elements ranges from 1.8 million to 5.1 million, averaging 3.5 million elements. The number of boundary layer meshes is 3, the layer decreasing ratio is 0.8, and the portion of edge size is 0.5 mm.

We note that the svSolver was extensively validated against in-vivo, in-vitro measurements, and intra-CFD group benchmark tests [27–29]. Previous validations studies showed excellent consistencies between aortic flow fields measured by svSolver and those by 4-D magnetic resonance imaging techniques [30, 31]. We believe that application of svSolver is suitable and provides credible quantitative visualization results of aortic flows.

### Post-Processing

After the simulations, we conducted post-processing on the hemodynamic variables. Two-dimensional slices perpendicular to the centerline of the aorta model were obtained at five specific locations (Fig. 2A). The first slice (Plane 0) was measured at the proximal site one diameter distanced from the two-dimensional slice above the annulus. The second slice (Plane 1) was measured at the proximal site one diameter distanced from the brachiocephalic artery. The third slice (Plane 2) was measured just before the brachiocephalic artery. The fourth slice (Plane 3) was measured right next to the left subclavian artery. The fifth slice (Plane 4) was measured at a location two diameters distanced from Plane 3.

In this study, we post-processed the CFD data and quantified hemodynamic metrics of clinical interest to characterize the hemodynamic differences among patient groups. Time-averaged wall shear stress (TAWSS) is one of the key hemodynamic parameters associated with many cardiovascular diseases thus being used for advanced treatment of cardiovascular disease. Low TAWSS is known to be associated with endothelial cell dysfunction, arterial plaque progression [32], vein graft failure, pulmonary hypertension [33], and aneurysm growth [34, 35]. Also, we investigated vorticity to study the dynamic characteristics of aortic flow in response to changes in the aortic root. Vorticity quantitatively measures how ‘swirly’ blood flows and we hypothesized that a straight blood flow pattern is more favorable than recirculating flows.

Wall shear stresses were time-averaged over one cardiac cycle, and area-averaged over the interested region near the sinus and the slice boundaries (Fig. 2B). We defined a region extending proximally from the STJ to a distance of one diameter to calculate the WSS in the aortic root (represented by the sky-blue color). Additionally, using the planes denoted as Plane 1–4 in Fig. 2A as references, we defined a region above and below each plane within a distance of 0.5 diameter. In this space, we measured the WSS for the proximal ascending aorta (red), distal ascending aorta (yellow), aortic arch (green), and descending aorta (blue).

The aortic arch angle was measured by first identifying the highest point of the centerline of the aorta (Point A) and drawing a tangent line from this point. Subsequently, a second line was created by moving one diameter parallel to the tangent line and determining two points where the centerline intersected with this line (Point B and C). The angle was then calculated between line AB and line AC (Fig. 2C).

Vorticities were calculated by taking spatial derivatives of the velocity components. Vorticity magnitudes were averaged over the two-dimensional slices and then averaged over the cardiac cycle at each five different locations along the centerline (Fig. 2A).

### Statistical Analysis

All data analyses were performed using SPSS statistics 25.0 (IBM, Armonk, NY, USA) and GraphPad Prism software. Data is presented as median and ranges. Not normally distributed data was compared between groups using the Mann–Whitney test, Fisher’s exact test and Kruskal–Wallis test. T-test was employed for normally distributed continuous variables between two groups. As appropriate, *p* < 0.05 was considered significant.

## Results

### Patients’ Baseline Characteristics and Cardiac CT Measurements

Baseline and surgical characteristics of the patients, along with cardiac CT measurements, are presented in Table 1. There were no significant baseline differences observed among the three groups. In Group A, the Z-score at the sinus of Valsalva was significantly higher compared to Groups B and C. Additionally, all patients in Group A had accompanying ventricular septal defect (VSD), while Group B did not.

**Table 1:** Baseline characteristics and cardiac CT measurements

	Group A (*n* = 5)	Group B (*n* = 5)	Group C (*n* = 5)	*P*-value	
				A-B	A-C
Patient and surgical characteristics					
Age, years	15 [9–18]	12 [10–14]	14 [8–18]	0.196	0.643
Male, n (%)	3 (60.0%)	4 (80.0%)	4 (80.0%)	1.000	1.000
Weight, kg	59.6 [42.0–82.0]	58.6 [44.5–80.5]	55.9 [44.2–84.2]	0.798	0.866
Height, cm	161.8 [139.0–172.3]	165.5 [149.3–169.1]	167.9 [133.5–177.3]	0.877	0.902
Body surface area, m^2^	1.6 [1.3–2.0]	1.6 [1.4–2.0]	1.7 [1.3–2.0]	0.907	0.926
Surgical variables					
Age at ASO, days	11.0 [4.0–37.0]	2.0 [1.0–10.0]	-	0.016	
Weight at ASO, kg	3.5 [3.3–4.7]	2.8 [2.6–3.4]	-	0.063	
Lecompte maneuver	5/5(100%)	5/5(100%)	-		
Transposition type, (%)				0.008	
TGA-IVS	0/5 (0.0%)	5/5 (100.0%)	-		
TGA-VSD	5/5 (100.0%)	0/5 (0.0%)	-		
Great artery positions, (%)				< 0.001	
Aorta right anterior to PA	0/5 (0.0%)	2/5 (40.0%)	0/5 (0.0%)		
Aorta anterior to PA	5/5 (100.0%)	3/5 (60.0%)	0/5 (0.0%)		
Aorta right posterior to PA	0/5 (0.0%)	0/5 (0.0%)	5/5 (100%)		
Aortic diameters (mm)					
Neo-aortic valve annulus	29.6 [22.0–34.3]	24.5 [22.4–27.3]	22.1 [19.5–25.7]	0.151	0.038
Neo-aortic sinus of Valsalva	38.7 [31.0–41.7]	28.4 [27.8–34.3]	30.2 [24.3–33.3]	0.016	0.008
Sino-Tubular Junction	29.8 [24.8–34.9]	23.3 [19.9–29.2]	26.0 [19.7–26.9]	0.025	0.024
Ascending aorta	25.0 [20.3–38.5]	23.9 [18.8–27.4]	24.2 [21.4–26.9]	0.215	0.340
Aortic Z-scores					
Neo-aortic valve annulus	4.4 [3.3–7.0]	3.1 [2.9–3.3]	1.7 [1.5–2.6]	0.059	0.009
Neo-aortic sinus of Valsalva	4.3 [3.8–6.5]	1.0 [0.3–3.6]	0.9 [0.4–1.8]	0.003	< 0.001
Sino-Tubular Junction	5.1 [0.4–6.3]	0.8 [-1.7–3.8]	1.2 [-1.2–1.8]	0.054	0.020
Ascending aorta	0.9 [-1.0–5.4]	0.2 [-1.8–2.0]	0.3 [-0.3–1.7]	0.246	0.337

Continuous variables were described as median and range

*TGA* Transposition of the great arteries, *ASO* Arterial switch operation, *IVS* Intact ventricular septum, *VSD* Ventricular septal defect, *PA* Pulmonary artery

### WSS and Vorticity Analysis among Three Groups

**Fig. 3:**
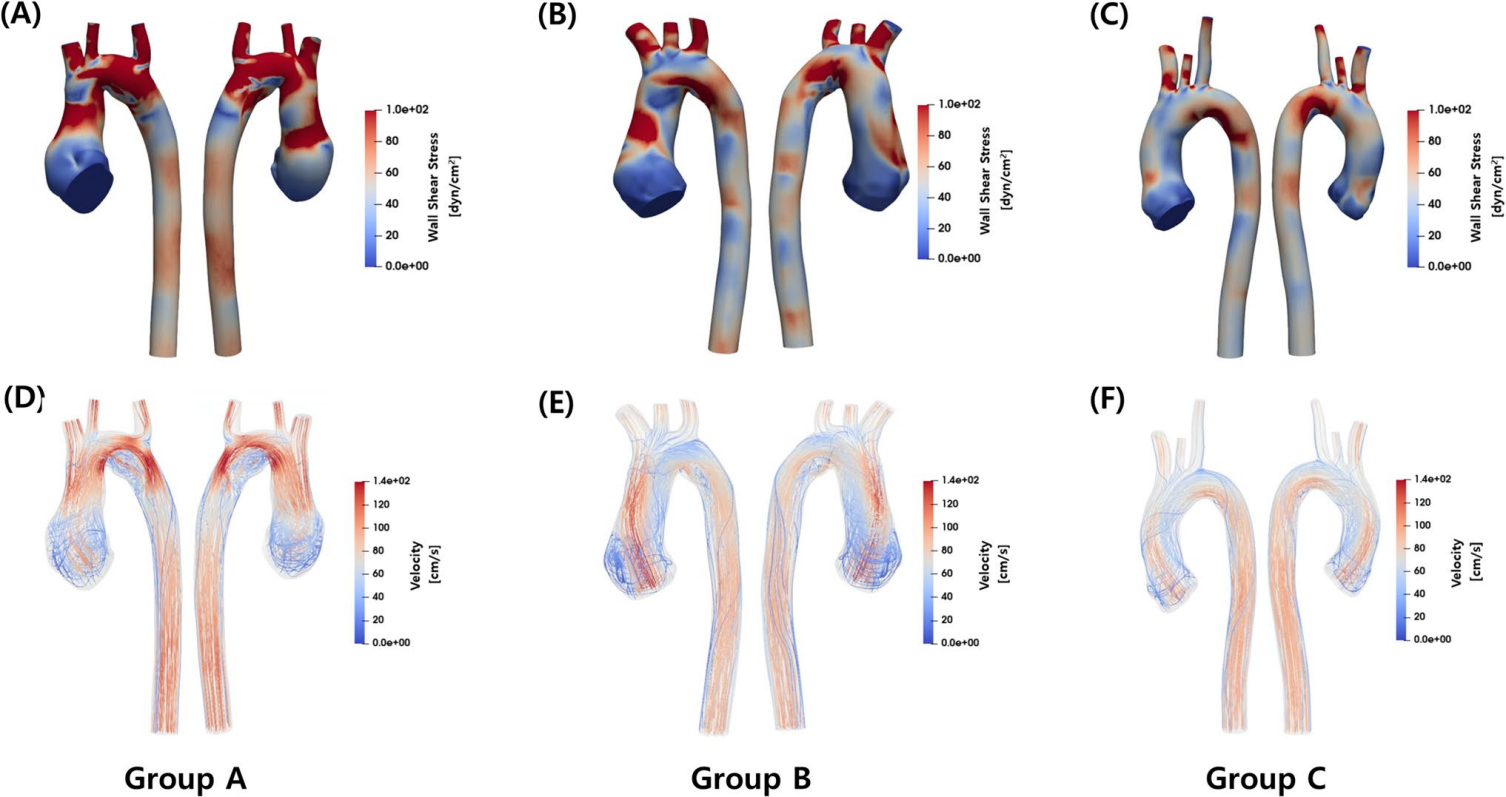
Peak systolic three-dimensional WSS visualization and streamlines in a representative case from each group. Panels (**A**-**C**) show Wall Shear Stress (WSS) images, where red areas indicate the maximum WSS. **A** is a representative case from the severe aortic root dilatation group in patients with TGA, with maximum WSS at the aortic root. **B** represents a case from the mild aortic root dilatation group in TGA patients, with maximum WSS at the distal ascending aorta. **C** is a normal control subject. Panels (**D**-**F**) depict 3D streamline images, with (**D**) showing a representative case of TGA with severe aortic root dilatation, (**E**) showing TGA with mild aortic root dilatation, and (**F**) showing the normal control group. WSS: Wall shear stress, TGA: transposition of the great arteries

Instantaneous three-dimensional WSS visualization and streamlines in a representative case from each group at systole phase are described in Fig. 3. The three-dimensional WSS visualization for entire patients is outlined in Supplementary Fig. 1, while focused images of vorticity measured during the systolic phase for each patient at the distal ascending aorta are described in Supplementary Fig. 2.

**Fig. 4:**
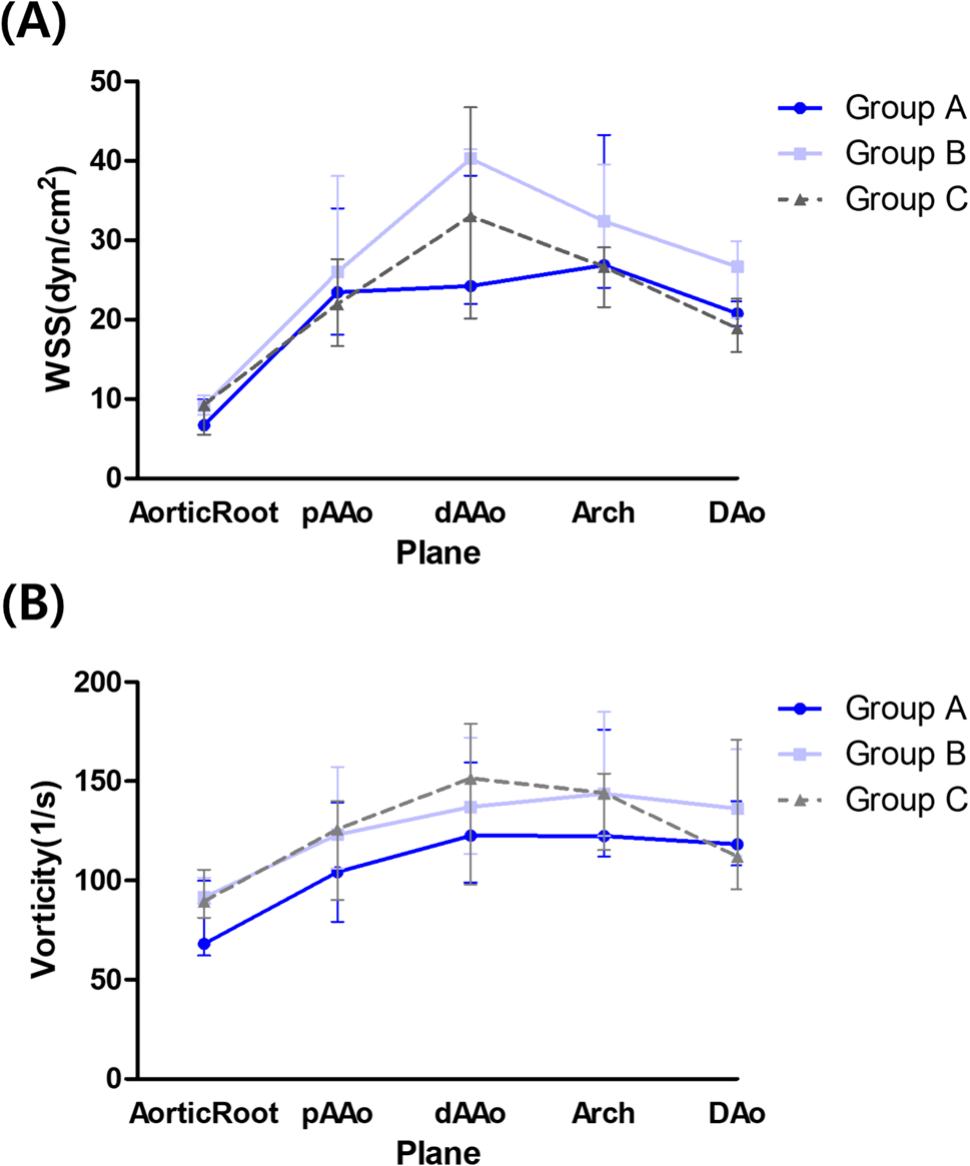
WSS and vorticity along the ascending aorta. **A** Patterns of wall shear stress (WSS) variation at five different aortic locations for each group. In the normal control group (Group C), WSS is lowest at the aortic root, peaks at the distal ascending aorta, and decreases thereafter. In the mild dilation group (Group B), WSS is higher than in Group C but follows a similar pattern. In the severe dilation group (Group A), the WSS peak at the distal ascending aorta is absent, and overall WSS is lower. **B** Patterns of aortic vorticity variation at five different locations for each group. Group C exhibits increasing vorticity until the distal ascending aorta, followed by a decrease. Groups A and B show different patterns, with the maximum vorticity observed in the aortic arch. Vorticity is lower in Group A compared to Groups B and C. (median, range). WSS: wall shear stress

The aortic WSS and vorticity along the entire thoracic aortic segments among three groups are summarized in Fig. 4. Figure 4A describes the patterns of WSS variation at five different locations. In the normal patient group (Group C), WSS displayed its lowest values at the aortic root, reaching its maximum at the distal ascending aorta, followed by a subsequent decrease. In the mild dilation group (Group B), WSS was higher than in Group C but followed a similar pattern. However, in the severe dilatation group (Group A), a distinct pattern emerged. Notably, at the distal ascending aorta, WSS was lower than in the normal patient group, while the maximum value was observed in the arch region. Figure 4B describes the pattern of vorticity variation at five different locations. In group C, vorticity exhibited the same pattern as WSS, increasing until the distal ascending aorta followed by a subsequent decrease. However, group A and B showed different patterns compared to group C, with the maximum vorticity observed at the aortic arch. In addition, in group A, there was a lower vorticity observed compared to group B and C.

**Fig. 5:**
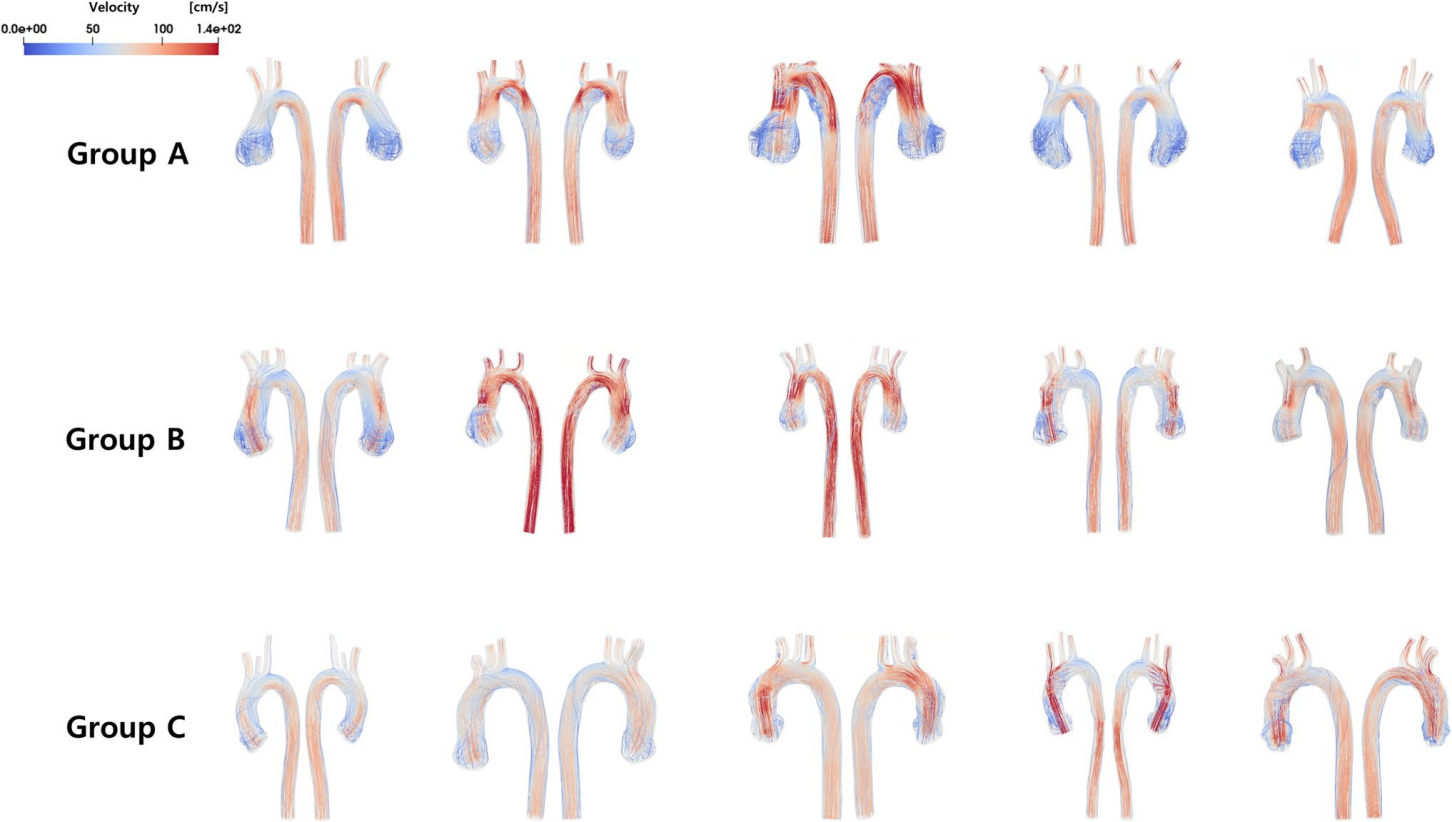
Streamlines analysis. The figure depicts streamlines for the entire cohort of 15 patients. In Group A, discernible recirculation flow occurs. Conversely, such recirculation is notably absent in Group C, while in Group B, its presence is not dominant

**Fig. 6:**
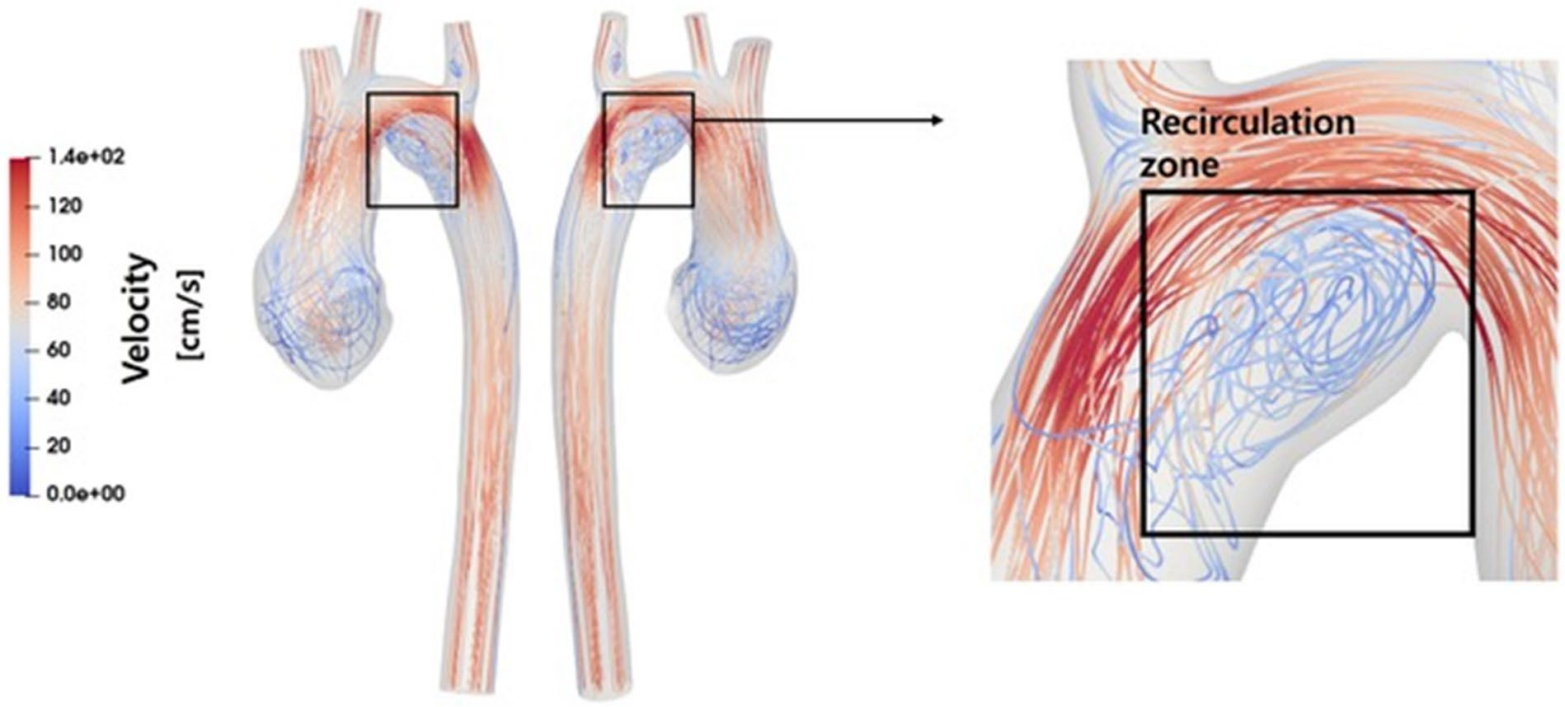
Visualization of Recirculation Flow Zone in a representative case from Group A. In Group A, a recirculation flow zone is present, which is not observed in other groups. This zone is located in the transverse aorta, as highlighted in the enlarged section on the right. The recirculation flow zone is characterized by swirling streamlines, indicating disturbed flow patterns and potential hemodynamic significance

Figure 5 shows the streamlines for the entire cohort of 15 patients. Notably, within Group A, a discernible recirculation flow was identified in the region between planes 2 and 3, as elucidated in Fig. 6 (transverse arch area), observed in the majority of patients. Conversely, such recirculation flow was notably absent in Group C. In Group B, while observed in a minority of patients, the presence of recirculation was not dominant.

### Comparison of Suggested Risk Factors for Aortic Root Dilatation among Three Groups

The differences among the three groups for the suggested risk factors for aortic root dilatation mentioned earlier are summarized in Table 2. Significant differences in anatomical parameters were observed among the groups. Group A exhibited significantly smaller aortic arch angles and preoperative great artery size discrepancies compared to Groups B and C. Additionally, all patients in Group A had accompanying VSD, which was absent in Group B. In Group A, disrupted WSS patterns and increased recirculation flow were observed, as detailed in Figs. 4, 5 and 6.

**Table 2:** Comparison of suggested risk factors for aortic root dilatation among three groups

	Group A (*n* = 5)	Group B (*n* = 5)	Group C (*n* = 5)	*P*-value		
				A-B	A-C	B-C
Demographic/clinical factors						
Male, n (%)	3/5 (60.0%)	4/5 (80.0%)	4/5 (80.0%)	1.000	1.000	1.000
Major coexisting cardiac lesions						
Ventricular septal defect	5/5 (100%)	0/5 (0.0%)	0/5 (0.0%)	0.008	0.008	
Taussig-Bing anomaly	0/5 (0.0%)	0/5 (0.0%)	0/5 (0.0%)			
Arch anomaly	0/5 (0.0%)	0/5 (0.0%)	0/5 (0.0%)			
Single coronary artery	1/5 (20%)	0/4 (0.0%)^†^	0/5 (0.0%)	1.000	1.000	
Bicuspid neo-aortic valve	0/5 (0.0%)	0/5 (0.0%)	0/5 (0.0%)			
Coronary anatomy^*^						
Usual (1LCx, 2R)	4/5 (80.0%)	3/4 (80.0%)^†^	5/5 (100%)	1.000	1.000	0.444
LCx from RCA (1L 2R,Cx)	0/5 (0.0%)	1/4 (20.0%)^†^	0/5 (0.0%)	0.444		0.444
Single LCA	1/5 (20.0%)	0/4 (0.0%)^†^	0/5 (0.0%)	1.000	1.000	
Factors associated with ASO						
Age at time of ASO, day	11.0[4.0 ~ 37.0]	2.0 [1.0 ~ 10.0]	-	0.016		
Pulmonary artery banding before ASO	0/5 (0.0%)	0/4 (0.0%)	-			
Great arteries size discrepancies	4/5 (80.0%)	0/4 (0.0%)	-	0.048		
Coronary transfer technique (Trap door technique)	4/4 (100.0%)^†^	4/4 (100.0%)^†^	-			
Post-ASO factors						
Neo-aortic regurgitation within 1 year after ASO	0/5 (0.0%)	0/4 (0.0%)^†^	-			
Arch angle(º)	72.3 [68.5–77.2]	76.6 [71.1–85.2]	97.3 [87.4–99.0]	0.132	< 0.001	0.002
Time interval from the operation to the last Cardiac CT, years	15 [9–18]	13 [10–14]	-	0.234		

Continuous variables were described as median and range

*TGA* Transposition of the great arteries, *ASO* Arterial switch operation, *LCx* Left circumflex artery, *RCA* Right coronary artery, *LCA* Left coronary artery, *CT* Computed tomography

^*^Coronary anatomy description according to the Leiden Convention

^†^There is 1 missing data

## Discussion

To the best of our knowledge, this study represents the first application of CFD to investigate altered hemodynamics in TGA patients who underwent ASO, stratified into severe dilatation, mild dilatation, and normal groups for comparative analysis. Our study identified distinct recirculation flow in the transverse aorta area in patients with severe aortic dilatation, associated with decreased WSS in that area. This pattern, unique compared to both normal conditions and mild dilation patients, suggests significant hemodynamic disturbances in severe dilatation. Aortic dilation leads to changes in WSS continuity, with increased WSS variation in mild dilation and decreased variation, and loss of the WSS peak in the distal ascending aorta in severe dilation. Mild dilation may increase blood flow and WSS, while severe dilation leads to decreased WSS due to the loss of effective blood flow delivery. Additionally, aortic vorticity changes were observed, with a decrease in vorticity variation between the aortic root, distal ascending aorta, and descending aorta as dilation increases (Group C: 62–39.4, Group A: 54.5–4.4, Group B: 45.5–0.8), and a tendency for increased vorticity in the descending aorta. Although the exact implications are not fully understood, these changes likely negatively impact vascular function especially affecting blood flow.

Previous studies reported that the specific geometry associated with the ASO, characterized by an acute angulation in the aortic arch, may contribute to increased aortic WSS, potentially playing a role in neo-aortic root dilatation [36, 37]. In our study, similar to previous research, significantly smaller aortic arch angles were observed in both severe and mild dilation groups compared to normal controls. However, the absence of significant differences in arch angles between severe and mild dilation groups indicates that factors beyond just the arch angle contribute to the extent of neo-aortic root dilation. Notably, we observed a distinctive presence of more pronounced recirculation flow in the transverse aorta area, specifically in severe dilation group. The formation of the recirculation zone in patients with severe dilation patient is believed to be influenced by the acute angulation of the arch and the dilatation of the aortic root, both of which deviate from the normal condition. From a hemodynamic perspective, as the dilation of the aortic root becomes more severe, it is likely to cause more pronounced bending of blood flow at the transverse aorta. This, in turn, leads to increased recirculation and a decrease in WSS at the transverse aorta. It is noteworthy that we do not observe such recirculated flow in the normal patients. This suggests that the geometry of the aortic arch of normal patients are optimized for hemodynamics, while the hemodynamics in patients with TGA deviates from this optimality.

Since we did not serially compare CT scans before and after the expansion of the neo-aortic root, establishing a clear cause-and-effect relationship is limited. However, given the distinctive features of acute arch angulation and recirculation in the severe dilatation group, it can be anticipated that preventing the severe dilation of the neo-aortic root would involve avoiding the occurrences of acute arch angulation and recirculation at the transverse aorta. Therefore, during ASO, it is vital to closely mimic the normal aortic arch shape. Furthermore, a heightened awareness of the aortic arch angle is essential, especially when there is a substantial difference in the size of the great arteries before surgery, as this condition increases the risk of forming recirculation zones.

Our evaluation of previously reported risk factors for neo-aortic root dilation revealed notable differences, particularly the higher prevalence of VSD, older age at ASO, and greater artery size discrepancy in the severe dilation group. These findings reinforce the significance of these factors in the progression of neo-aortic root dilation and underscore the need for careful preoperative assessment and postoperative monitoring.

This study employed CFD to conduct blood flow visualization through streamlines and evaluate hemodynamic parameters including WSS and vorticity. WSS is defined as the viscous shear stress of blood flow acting tangentially to the vessel wall. Vorticity is a vector field variable derived from the velocity vector, representing the rotationality of flows. Elevated WSS regions have been linked to significant histological alterations in the vessel wall of the ascending aorta in patients with bicuspid aortic valves [38]. Moreover, abnormal WSS has been implicated as a potential contributing factor to vessel dilation and the development of aneurysms, such as those in the thoracic aorta and intracranial regions [34, 35]. Given the results from previous studies, it was anticipated that WSS would be highest in Group A. However, in Group B with mild dilatation, there was an unexpected observation of higher WSS and vorticity compared to Group A. This might be attributed smallest diameter of the group B among all groups, because the analysis was conducted under conditions where the difference in cardiac output was not substantial across all patient groups. From the analytical solution of Poiseuille flow under the assumption of a steady, laminar flow in a circular cylinder, we can evaluate the trend of the wall shear stress while changing the diameter of the vessel as ${\text{WSS}}_{\text{Poiseuille}}=\frac{32\mu Q}{\pi {D}^{3}}$, where $\mu$ is the dynamic viscosity of blood, Q is the blood flow rate, and D is the diameter of the vessel [39]. When the flow rate is fixed, the WSS is inversely proportional to the diameter to the third power. Therefore, the smaller diameter in the Group B could lead to the higher measurements of WSS. Furthermore, from a clinical perspective, after the ASO for TGA, the creation of acute angulation in the aortic arch is associated with increased WSS. This elevated WSS may facilitate the progression of vessel dilation. As dilation progresses, the expectation is for the emergence of blood flow recirculation zones, which lead to localized regions of reduced WSS. Consequently, this phenomenon explains the observation of lower WSS in Group A compared to Group B.

The vorticity of Group A is smaller than that in Groups B and C. We believe this is mainly due to a decreased velocity gradient at the inlet of the model resulting from severe dilation, attributed to mass conservation when the cardiac output remains largely unchanged. The origin of larger vorticity can be attributed to a larger mean shear in the upstream region, which strengthens the shear layer instability, breaking the mainstream large-scale flows into smaller eddies. While we are providing fluid mechanics theory-based explanations for observed hemodynamics, a further fundamental study is warranted for supporting this trend.

Additionally, we focused on not just the absolute values but also the changing patterns of WSS and vorticity. In normal population, WSS is lowest at the aortic root, gradually increasing and reaching a peak at the distal ascending aorta, and then decreasing as it continues into the descending aorta, exhibiting a continuous pattern. However, in cases of severe vessel dilation, we observed a discontinuous pattern where WSS decreased in the distal ascending aorta and exhibited the maximum value in the aortic arch region. The causal relationship remains unclear, and there is an ongoing debate regarding the risk factors influencing aortic root dilation. Therefore, further larger-scale and consecutive studies are required in the future to provide more definitive insights.

There are limitations to the present study. This is a retrospective study and is therefore subject to limitations inherent to the design. A serial of measurements from birth to adulthood was not available in all patients. In particular, for records immediately after birth, as cardiac CT scans were not frequently performed, we referred to echocardiography records. For records predating the use of digital recording systems, mentions of the size difference between the aorta and pulmonary artery existed on the records, but precise size measurements were not possible, making it impossible to calculate the z-score. Furthermore, due to the limited number of patients in each group, it is challenging to establish statistically significant correlations between the groups. Hence, to establish a more definitive relationship, further research with an expanded cohort of patients is warranted. Additionally, this study did not compare serial CT images taken before and after the occurrence of aortic root dilatation; rather, only used CT images taken between the ages of 8 and 18 for CFD analysis. As a result, there are limitations regarding the potential ambiguity in establishing causality. Despite the limitations, it is noteworthy that our study has identified recirculation blood flow and an abnormal WSS pattern in severe aortic root dilation in patients with TGA after ASO which can be important to understand the hemodynamics for these patients.

In conclusion, patients with severe aortic root dilatation exhibit a distinct pattern of WSS variation, differing from that of normal and mildly dilated patients. This unique pattern is particularly evident in the transverse aorta area.

## Supplementary Information

Below is the link to the electronic supplementary material.

Supplementary Fig. 1 Peak systolic three-dimensional WSS visualization for the entire cohort of 15 patients. The red area indicates the maximum WSS. (PNG 481 kb)

High resolution image (TIF 3455 kb)

Supplementary Fig. 2 Focused images of vorticity at the systolic phase for each patient. Focused images of vorticity measured during the systolic phase for each patient at Plane 2, as illustrated in Fig. 2A. The flow vorticity is color-coded based on the strength of the streamwise vorticity component. (PNG 1149 kb)

High resolution image (TIF 8024 kb)

## Data Availability

The data that support the findings of this study are available from the corresponding author upon reasonable request.

## Abbreviations

TGA: Transposition of the great arteries
ASO: Arterial switch operation
BSA: Body surface area
CFD: Computational fluid dynamics
WSS: Wall shear stress
TAWSS: Time-averaged wall shear stress
CT: Computed tomography
STJ: Sino-tubular junction
VSD: Ventricular septal defect
