# Supplementary figures and images for: Hemodynamic Analysis in Aortic Dilatation after Arterial Switch Operation for Patients with Transposition of Great Arteries Using Computational Fluid Dynamics
Source: J Cardiovasc Transl Res. 2024 Sep 25;18(1):79–90. doi: 10.1007/s12265-024-10562-2 (PMC11885326; doi:10.1007/s12265-024-10562-2)

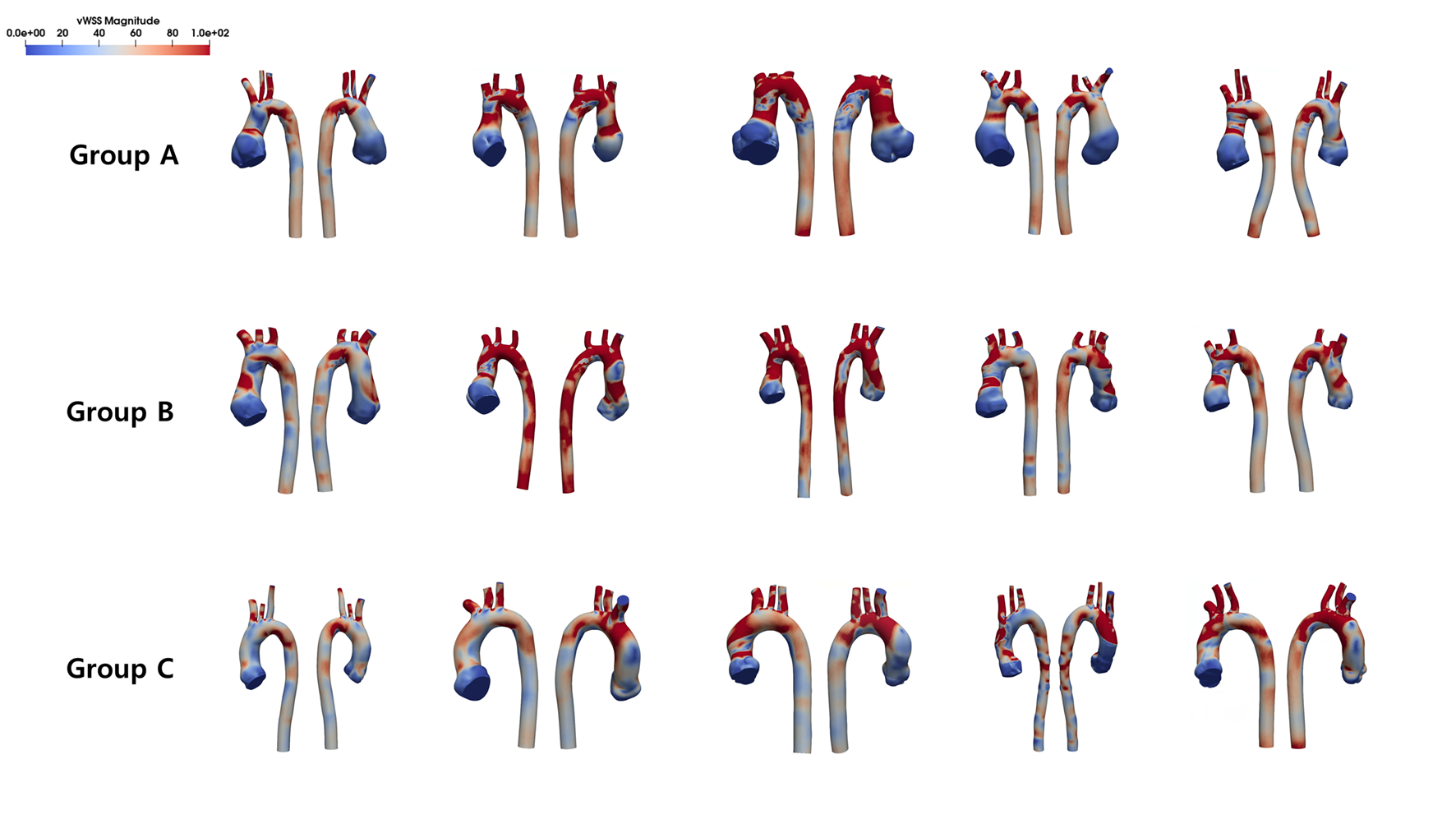

Supplement: Supplementary file 1 — Peak systolic three-dimensional WSS visualization for the entire cohort of 15 patients. The red area indicates the maximum WSS. (PNG 481 kb) [file 12265_2024_10562_Fig7_ESM.png]

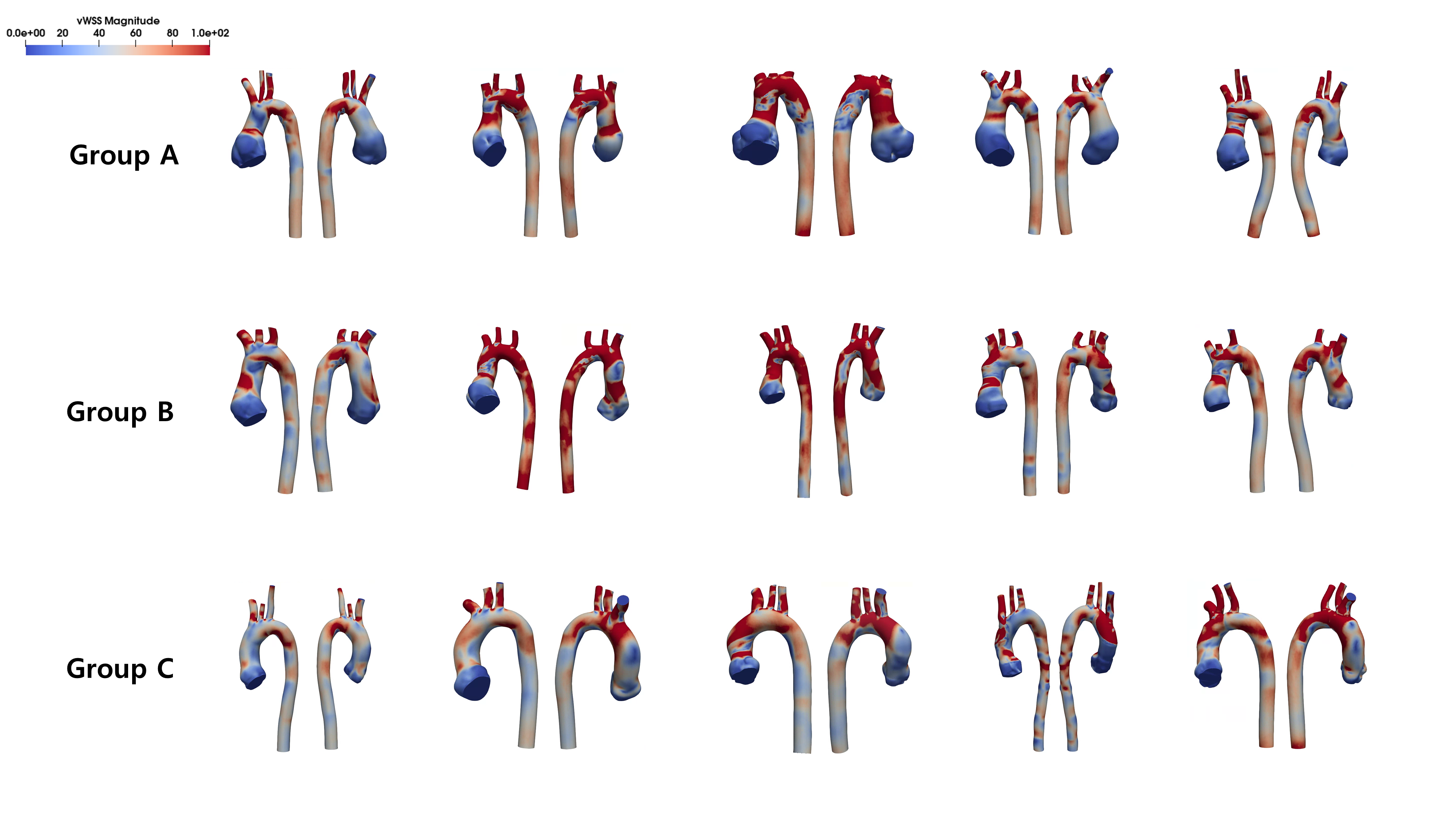

Supplement: Supplementary file 2 — High resolution image (TIF 3455 kb) [file 12265_2024_10562_MOESM1_ESM.tif]

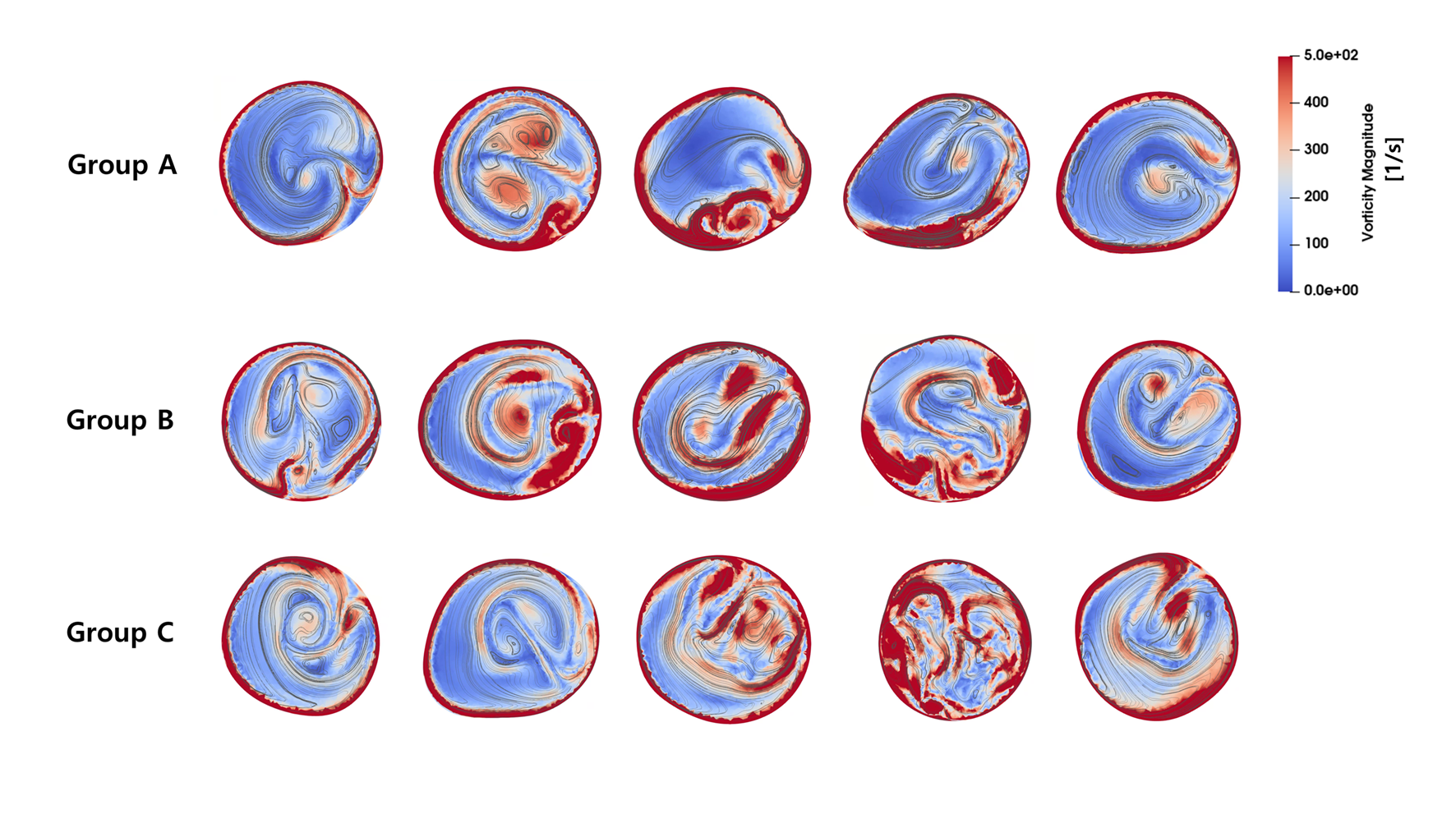

Supplement: Supplementary file 3 — Focused images of vorticity at the systolic phase for each patient. Focused images of vorticity measured during the systolic phase for each patient at Plane 2, as illustrated in Fig. 2A. The flow vorticity is color-coded based on the strength of the streamwise vorticity component. (PNG 1149 kb) [file 12265_2024_10562_Fig8_ESM.png]

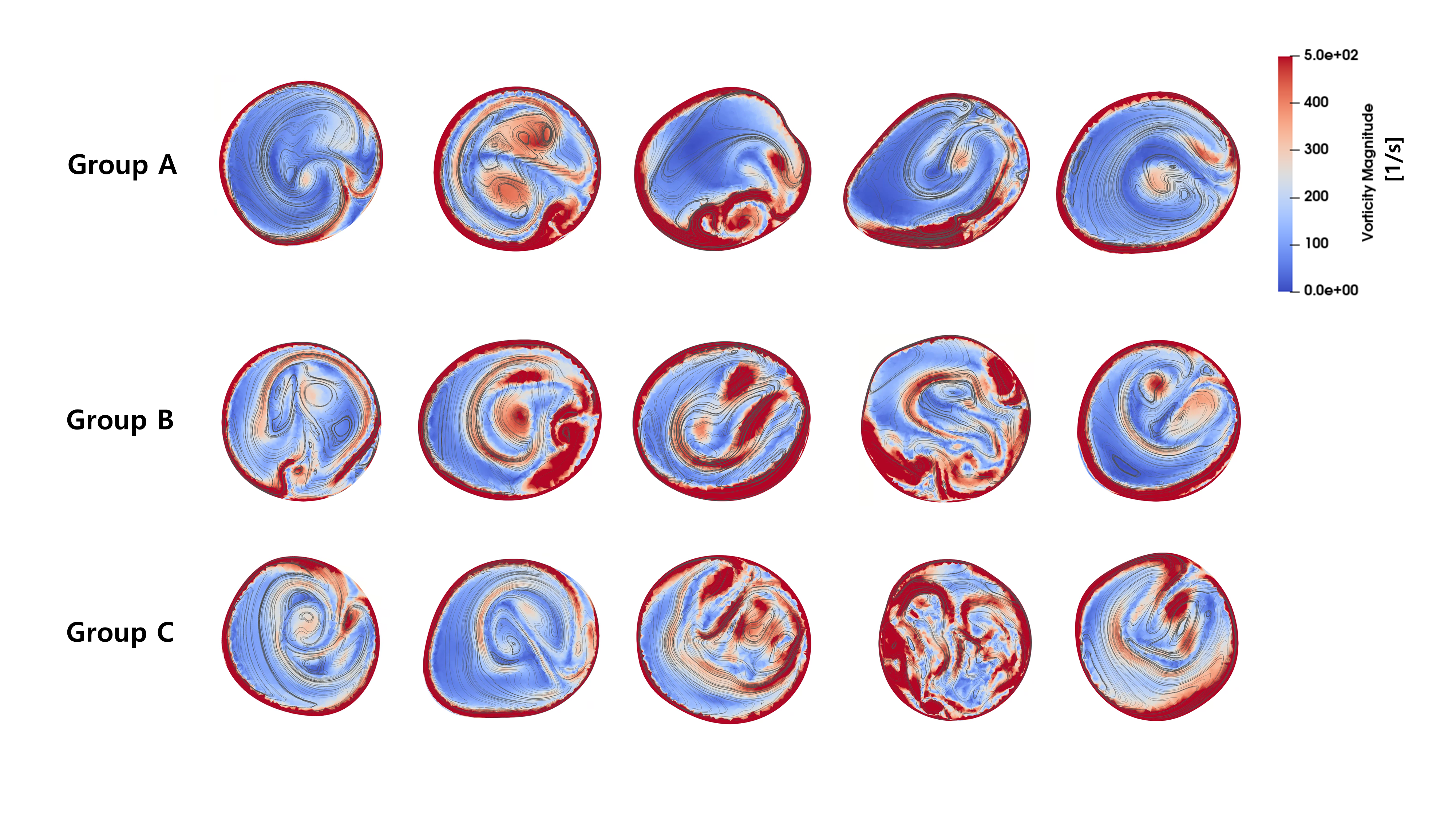

Supplement: Supplementary file 4 — High resolution image (TIF 8024 kb) [file 12265_2024_10562_MOESM2_ESM.tif]
